# Genetic diversity of melon aphids *Aphis gossypii* associated with landscape features

**DOI:** 10.1002/ece3.4181

**Published:** 2018-05-24

**Authors:** Zhaoke Dong, Yifan Li, Zhiyong Zhang

**Affiliations:** ^1^ Beijing Key Laboratory of New Technology in Agricultural Application National Demonstration Center for Experimental Plant Production Education Beijing University of Agriculture Beijing China; ^2^ College of Plant Protection Northwest A &F University Yangling China

**Keywords:** 2b‐RAD sequencing, landscape genetics, SNP loci, spring migration, watermelon

## Abstract

Despite increasing evidence that landscape features strongly influence the abundance and dispersal of insect populations, landscape composition has seldom been explicitly linked to genetic structure. We conducted a genetic study of the melon aphid, *Aphis gossypii*, in two counties of Beijing, China during spring migration using samples from watermelon. We performed aphid genetic analysis using restriction site associated DNA sequencing (2b‐RAD) and investigated the relationship between land cover and the genetic diversity. The percentage area of land cover (cropland, vegetable, orchard, grassland, woodland) was quantified in each particular scale (ranging from 0.5 km to 3 km) and was used as a predictor variable in our generalized linear models. We found a moderate level of genetic differentiation among nine sampled populations. Geographic distance and genetic distance were not significantly associated, indicating that geographic location was not a barrier to migration. These nine populations could be clustered depending on their level of genetic diversity (high and low). The genetic diversity (Shannon’s information index) was positively correlated with grassland at the spatial scales of 1 and 2 km and negatively with orchard and vegetable at 0.5 and 1 km. Genetic diversity was best predicted by the grassland + orchard + vegetable model at a spatial scale of 1 km. Based on the method of relative weights, orchard land had the greatest relative importance, followed by grassland and vegetable land, in that order. This study contributes to our understanding of the genetic variation of aphids in agricultural landscapes.

## 1 INTRODUCTION

Understanding how genetic variation is partitioned across the genome, both within and among populations, is a fundamental issue in ecological and evolutionary genetics (Catchen et al., 2013). Landscape genetics is an important emerging research topic that integrates landscape patterns with population genetics (Holderegger & Wagner, 2006). Studies in landscape genetics primarily evaluate the role of habitat loss and fragmentation in shaping the population genetic structure of a particular species, for example, bird, frog, or bee (Herrmann, Westphal, Moritz, & Steffan‐Dewenter, 2007; Johansson, Primmer, Sahlsten, & Merilä, 2005; Lindsay et al., 2008). Landscape variables are typically analyzed as putative barriers to the movement of individuals (Hemme, Thomas, Chadee, & Severson, 2010; Keyghobadi, Roland, & Strobeck, 1999), but land cover data have been seldom used (Perez‐Espona, McLeod, & Franks, 2012). Moreover, analyses of the impact of agricultural landscape features on the population genetics of insect pests are sparse. Here, we assess the effect of land cover on the genetic diversity of an insect pest using the melon aphid, *Aphis gossypii* Glover, as a model.

The melon aphid is distributed worldwide and is extremely polyphagous. It causes serious damage to melon, cotton and hundreds of other plant species (Blackman & Eastop, 2007). This aphid also has a complex life history. In regions with severe winters, *A. gossypii* has a complete life cycle (or holocycle). It lays eggs in autumn on a primary host (e.g., hibiscus), and in the spring, it migrates to secondary host plants (Zhang & Zhong, 1982). In areas with mild winters, it reproduces asexually throughout the year (Agarwala & Das, 2007; Fuller, Chavigny, Lapchin, & Vanlerberghe‐Masutti, 1999). *A. gossypii* is an important pest species of melon crops in the Beijing area. In recent years, the forest coverage in Beijing has greatly increased, and its urban agriculture is developing rapidly. Land use has changed, and the areas of cropland have decreased while the areas of woodland have increased. It is important to understand the relationship between the population genetics of aphids and landscape features.

The genetic variation of melon aphids can be attributed to many factors. One of the main factors is host plant species. The population genetics of *A. gossypii* is well documented to be affected by host plant species (Carletto et al., 2009; Charaabi et al., 2008). The population of *A. gossypii* often shows some degree of host specialization. Several host races are recognized the world over on congeneric host plants (Agarwala & Choudhuri, 2014) and on cultivated plants (Guldemond, Tigges, & De Vrijer, 1994). Reproductive mode is another factor influencing genetic diversity. Aphids reproducing by parthenogenesis have low genetic diversity at local and worldwide scales (Carletto et al., 2009; Chen et al., 2013). In contrast to the populations with an incomplete life cycle (or anholocycle), holocycle populations sexually reproduce. Evidence for high genetic diversity associated with sexual reproduction of *A. gossypii* has been found in China (Wang et al., 2016), France (Thomas, Boissot, & Vanlerberghe‐Masutti, 2012), and Iran (Razmjou, Vorburger, Moharramipour, Mirhoseini, & Fathipour, 2010). In addition, other factors such as insecticide selection pressure and competition can also affect genetic diversity (Brévault, Carletto, Linderme, & Vanlerberghe‐Masutti, 2008; Brévault, Carletto, Tribot, & Vanlerberghe‐Masutti, 2011; Carletto, Martin, Vanlerberghe‐Masutti, & Brévault, 2010; Fuller et al., 1999).

The effects of landscape features on aphid dispersal have been documented (Angelella, Holland, & Kaplan, 2016; Gilabert et al., 2017). Thomas, Vanlerberghe‐Masutti, Mistral, Loiseau, and Boissot (2016) conducted demo‐genetic analyses of aphid populations on aphid‐resistant plants during growth seasons and found that plant resistance changed the genetic structure of the aphid populations. Factors that modify the dispersal of founders may also modify the genetic composition of the population. Overall, the host colonization of *A. gossypii* likely could affect their population genetics. It has been suggested that host colonization of *A. gossypii* often occurs locally (Agarwala & Choudhuri, 2014; Carletto et al., 2009).

The landscape genetics of *A. gossypii* would provide new insights into host plant colonization. We conducted a landscape scale study on the population diversity of *A. gossypii* based on SNP loci in the genome. The advantage of a SNP study compared to using microsatellite data is that one can obtain more information. SNP data have the potential to identify both historical population structure and recent colonization history (Catchen et al., 2013). We focused on the spring migration of *A. gossypii* (founding individuals) to address the following questions: 1) Can genetic analysis reveal landscape aspects of *A. gossypii* colonization? 2) Are landscape features associated with the genetic diversity of *A. gossypii* colonizing the same host?

## 2 MATERIAL AND METHODS

### 2.1 Sampling

**Figure 1:**
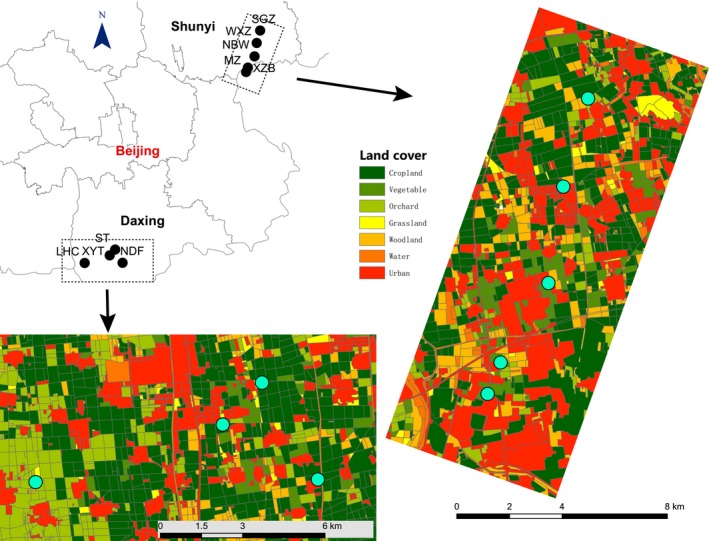
Sample localities of aphid populations in the vicinity of Beijing, China

Sampling was conducted in the Beijing area of northern China (Figure 1). There were nine sites located in Daxing and Shunyi counties. Daxing is located south of Beijing, and Shunyi is northeast of Beijing. These two counties are in plain areas dominated by cultivated land. The sampling protocol consisted of collecting populations from as many watermelon plants as possible from different sites with a variety of landscape complexity.

Sampling was carried out in the early growth season, when aphids were migrating from primary hosts to the secondary host (melons). All melon aphids were sampled in greenhouses, because the spring melon crop is usually produced in greenhouses. We sampled 3–5 greenhouses at each location. In each greenhouse, we sampled five clones randomly by collecting leaves containing aphids. In each clone, only one alate aphid was used for further analysis. We randomly chose 10 individuals per location to represent the population of that location. Sample sizes for 2b‐RAD sequencing are listed in Table 1. A total of 90 aphids from nine locations were used for analysis of the population structure. All aphid samples were preserved in 95% ethanol at room temperature for DNA testing. We also obtained the sequence of the mitochondrial gene COI that had the best match with the COI sequence of *A. gossypii* in NCBI database. All samples (including those used for DNA analysis) in this study were deposited at our laboratory.

**Table 1:** Sample collection data and genetic diversity^a^

County	Sites (village)	Population	Sampling date	N	%poly	I	Ho	He	F
Daxing	Lihuacun	LHC	May 9‐26	10	25.96	0.156	0.180	0.107	−0.441
Daxing	Nandunfa	NDF	May 9‐26	10	69.43	0.306	0.153	0.181	0.385
Daxing	Shitong	ST	May 9‐26	10	78.54	0.361	0.172	0.210	0.286
Daxing	Xiyitang	XYT	May 9‐26	9	78.60	0.396	0.178	0.236	0.328
Shunyi	Mazhuang	MZ	May 24‐31	7	60.78	0.335	0.214	0.209	0.126
Shunyi	Nanbeiwu	NBW	May 24‐31	10	28.57	0.157	0.180	0.107	−0.452
Shunyi	Songgezhuang	SGZ	May 24‐31	10	28.26	0.165	0.179	0.112	−0.452
Shunyi	Wangxinzhuang	WXZ	May 24‐31	10	66.34	0.379	0.259	0.233	0.286
Shunyi	Xiaozhubao	XZB	May 24‐31	10	62.42	0.298	0.169	0.182	0.149

a N = sample size, %poly = percentage of polymorphic loci, I = Shannon’s information index, Ho = observed heterozygosity, He = expected heterozygosity, F = fixation index.

The distance between any two sample sites was at least 1 km. GPS coordinates were recorded using a Magellan^®^ handheld GPS receiver, a latitude‐longitude projection and WGS84 datum. Sampling locations were further confirmed by consulting topographical maps of the study area and using Google Earth.

### 2.2 2b‐RAD sequencing and genotyping

Genomic DNA was isolated using the DNeasy Tissue Mini Kit (Tiangen) following the manufacturer instructions. A total of 90 aphids were genotyped for SNP loci. 2b‐RAD sequencing and genotyping were outsourced to Shanghai OE Biotech Ltd. (Shanghai, China). The 2b‐RAD libraries were prepared following the protocol developed by Wang, Meyer, McKay, and Matz (2012). In brief, DNA from each sample was digested using BsaXI and then verified and separated on agarose gel. Next, library‐specific adaptors and the digestion products were linked with T4 DNA ligase. Ligation products were amplified by PCR, and the target band was excised from a 2% agarose gel. At last, sample‐specific barcodes were introduced by PCR with platform‐specific barcode‐bearing primers. The PCR products were purified using the QIAquick PCR purification kit (Qiagen, Germany) and then pooled for sequencing using the Illumina Hiseq2500 platform (Illumina, San Diego, CA).

Quality filtering was conducted as follows: raw reads were trimmed to remove adaptors, and the terminal 2‐bp positions were discarded to eliminate artifacts that might have arisen by ligation. Reads contained no restriction site or long homo‐polymer regions (>10 bp). Ambiguous bases (N) or reads of low quality (>10 bp with quality less than Q20) were removed. SNPs were determined, and genotypes were called using a maximum‐likelihood statistical model implemented in the software Stacks v1.32 (Catchen, Amores, Hohenlohe, Cresko, & Postlethwait, 2011).

### 2.3 Data analysis

#### 2.3.1 Population genetic analyses

The genotype data contained information for each locus and each individual. We used MEGA 7 to set the coverage at 95% and then output the selected loci for further analysis. All the genetic analyses were performed in GenALEx 6.5 (Peakall & Smouse, 2006, 2012). The genetic diversity of sampling locations was quantified using allele frequency, Nei’s unbiased heterozygosity, and Shannon’s information index. Shannon’s information index for information theory has often been used in population genetics. It offers statistical properties useful for measuring biological information (Peakall & Smouse, 2012; Sherwin, Jabot, Rush, & Rossetto, 2006). Therefore, we used Shannon’s information index to represent the genetic diversity. We estimated genetic variation (Fst) among all populations and between each pair of populations. To determine the proportion of genetic variation that could be attributed to differences between sampling sites, hierarchal analyses of molecular variance (AMOVAs) were performed. The correlation between genetic distance and geographic distance were analyzed using Mantel tests (10,000 permutations) which is the most widely used statistical method in landscape genetics’ studies. Principal coordinates analysis (PCoA) was used to find and plot the major pattern within a genetic distance matrix dataset.

#### 2.3.2 Influence of landscape features on genetic diversity

Land cover maps of sampling sites were obtained from a digital map provided by the ZiYuan‐3 survey satellite (ZY‐3) with 5 m resolution. The landscape structure was analyzed using ArcGIS 10 (ESRI, Redlands, CA, USA). Land covers were classified into seven general categories, including cropland, vegetable, orchard, grassland, woodland, water, and urban. Cropland was categorized as land primarily being cultivated with crops such as winter wheat. The vegetable category included protected fields, for example, greenhouses, in which vegetables were usually planted. The orchard category had perennial crops, such as orchard trees. The grassland category was herb‐dominated land. Woodland included all forest and shrub land excluding the trees in residential sites. Water represented wetland and open water. Urban represented human architecture, roads, etc. For each of the sampling sites, landscape composition was estimated in four circular sectors (0.5, 1, 2, and 3 km radii), representing a nested set of landscape sectors at four spatial scales. The percentage of each category was measured within each landscape circle.

Generalized linear model regression (GLM) and correlation analyses were performed using the statistical program R version 3.4.0 (R Core Team 2017). Genetic data, such as Shannon’s information index, the percentage of polymorphic loci and expected heterozygosity were highly correlated. Only observed heterozygosity had a weak correlation with all the other indices. We used Shannon’s information index as a response variable and landscape features as predictor variables. The landscape variables were the percentages of cropland, vegetable, orchard, grassland, and woodland, all of which were belonging to vegetation cover. Each spatial scale was analyzed separately. We used the all‐subsets regression to select the predictor variables that best explained the model (Kabacoff, 2015). The all‐subsets regression was achieved using the package “leaps” (Thomas Lumley based on Fortran code by Alan Miller, 2017). We fit the models based on the all‐subset regression results. For all the scales, we chose the best model among different scales by adjusted r^2^, P‐value, and AIC. We used a new method called relative weights to assess the relative importance of predictors. The method closely approximates the average increase in R‐square obtained by adding a predictor variable across all possible submodels (Kabacoff, 2015).

## 3 RESULTS

### 3.1 Bioinformatics and filtering of data

Sequencing resulted in an average of 6,927,570 bp reads per individual. The percentage of high quality of reads was above 70% of the total reads in the libraries of the 90 individuals. The SNP loci number was 10,685, which could genotype at least 72 individuals (80% of samples). We removed four individuals (3 in MZ and 1 in XYT) because of their low‐quality reads and obtained a total of 6,280 loci, which scored 95% coverage.

### 3.2 Genetic diversity of aphid populations

The percentage of polymorphic loci ranged from 25.96% (LHC population) to 78.60% (XYT population). Shannon’s information index values across loci ranged from 0.156 (LHC population) to 0.396 (XYT population). The mean observed heterozygosity (Ho = 0.187) was similar to the mean expected heterozygosity (He = 0.175) in the total number of populations (Table 1). The reduction in genetic variation in the LHC and NBW populations was evident in their percentages of polymorphic loci, which were much lower than XYT and WXZ populations.

There was a moderate level of genetic differentiation among populations. Pairwise Fst values between the populations ranged from −0.042 to 0.310 (Table 2). The Mantel test result showed that no significant correlation was found between genetic and geographic distances across the nine populations (*Z* = −7.248, *r* = −0.074, *p* = 0.276).

**Table 2:** Pairwise comparison of genetic distance (Fst) and geographic distance (km) among aphid populations

	LHC	NDF	ST	XYT	MZ	NBW	SGZ	WXZ	XZB
LHC		0.206	0.155	0.094	0.038	0.001	0.012	0.310	0.171
NDF	10.306		−0.042	0.001	0.119	0.170	0.125	0.155	0.030
ST	9.027	4.088		−0.020	0.075	0.123	0.086	0.131	0.011
XYT	7.149	4.010	2.098		0.014	0.072	0.045	0.105	0.006
MZ	67.910	61.698	59.940	62.024		0.035	0.032	0.175	0.080
NBW	72.661	66.528	64.741	66.823	4.843		−0.007	0.292	0.137
SGZ	79.087	73.272	71.351	73.420	11.871	7.145		0.268	0.108
WXZ	75.859	69.925	68.052	70.126	8.421	3.710	3.455		0.134
XZB	69.116	62.943	61.169	63.251	1.268	3.586	10.605	7.154	

Above diagonal; genetic distance among populations as measured by Fst.

Below diagonal; geographic distance between populations (km).

**Figure 2:**
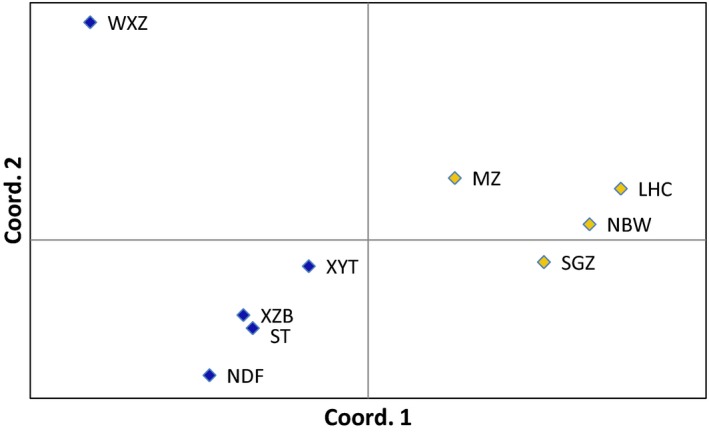
Principal coordinate analysis based on the genetic distance matrix of Fst values. Population codes are given in Table 1. Colors within the diamond: blue, high genetic diversity group; yellow, low genetic diversity group

According to the results of AMOVA, the majority (89%) of the genetic variation was attributable to variation within populations, whereas approximately 11% of the variation was attributable to variation between populations. However, no variation was attributed to variation among regions (Daxing county and Shunyi county) (Table 3). The PCoA result (Figure 2) showed a high–low pattern of genetic structure. The first and second axes explained 69.04% and 23.95% of the overall variance, respectively. We, therefore, divided the populations into two groups: higher genetic diversity group (WXZ, XYT, ST, XZB, NDF), and lower genetic diversity group (MZ, SGZ, NBW, LHC). The AMOVA result showed that 11% of the variation was attributable to variation among these two groups (Table 3).

**Table 3:** AMOVA analysis testing the partitioning of genetic variation across populations, geographic regions (Daxing and Shunyi) and two groups including a high genetic diversity group and a low genetic diversity group based on STRUCTURE analysis

Source of variation	*df*	SS	% Variation	*P*‐value
Global analysis				
Among region	1	1490.994	0	0.971
Among population	7	13498.768	11%	0.001
Among individual	77	43315.367	0%	0.001
Hierarchical AMOVA (K = 2)				
Among group	1	7216.514	11%	0.001
Among population	7	7773.224	4%	0.001
Among individual	77	43315.367	0%	0.001

### 3.3 Genetic diversity and their relationship with landscape feature

Shannon’s index was positively correlated with grassland at the spatial scales of 1 and 2 km and negatively with orchard at 0.5 and 1 km. Shannon’s index was best predicted by the grassland + orchard + vegetable model at a spatial scale of 1 km (Table 4), having the lowest AIC score of any model at the four spatial scales examined. The correlation between Shannon’s index with vegetable land was marginally significant in this model (*p* = 0.066). The total amount of variance accounted for by this model (*R*‐square = 0.796) was divided among the predictor variables. Orchard land accounted for 57.80% of the *R*‐square, grassland accounted for 27.72%, and vegetable land accounted for 14.48%. Based on the method of relative weights, orchard land had the greatest relative importance, followed by grassland and vegetable land, in that order.

**Table 4:** The best model of GLM regression of genetic diversity (I, Shannon’s information index) related to landscape features. Landscape variables included percentage of cropland areas (cropland), percentage of vegetable areas (vegetable), percentage of orchard areas (orchard), percentage of grassland areas (grassland), and the percentage of woodland areas (woodland) at each scale^a^

Scale	Model	Adjusted *r* ^2^	*p* value	AIC
0.5 km	−0.0021 × Orchard	0.279	0.083	−15.399
**1 km**	−**0.0042 × Vegetable − 0.0033 × Orchard** b ** + 0.036 × Grassland** b	**0.674**	**0.035**	−**21.576**
2 km	0.060 × Grassland^b^	0.353	0.054	−15.399
3 km	−0.0028 × Orchard	0.105	0.206	−13.458

a Bold indicates the best overall model. Intercept of models are omitted.

b Significance of variables is indicated as follows: *p *< 0.05.

## 4 DISCUSSION

### 4.1 Genetic diversity of *A. gossypii* in melon growing areas

We completed genetic analysis of *A. gossypii* samples collected from watermelon in the Beijing area at landscape scales. We found evidence for moderate population differentiation at different sample sites with landscape features associated with the genetic variation.

Samples of *A. gossypii* populations were collected from watermelon on which reproduction was via apomictic parthenogenesis. The sampling period was during the establishment of the spring migration population. Early season individuals on melon were alates, and so the entire potential colonizing population was sampled. Host plant pressure on aphids occurs in melons and aphid‐resistant melon cultivars and could alter the genetic structure of *A. gossypii* (Thomas et al., 2012, 2016). In the present study, there was no known commercial aphid‐resistant melon varieties planted in the research area. Most of the watermelon cultivars in the study area were “Jingxin.” We therefore assume that any host plant pressure on the different *A. gossypii* samples was similar across the study area. We therefore excluded potential host plant effects and focused on the possibility that the different surrounding habitats could influence the genetic diversity of the melon aphid populations in greenhouses.

A recent genetic study of *A. gossypii* in China, based on microsatellite and mitochondrial DNA data, showed that aphids were genetically clustered into a western group and an eastern group (Wang, Yang, Lu, Zhou, & Wu, 2017). Their study had a large geographic scale, so a geographic effect was expected. In the smaller landscape scale of the present study, we found no geographic isolation. Most aphids are weak fliers, and their dispersal among secondary hosts is aided by wind (Dixon & Howard, 1986; Taylor, Woiwod, & Taylor, 1979). The genetic analysis showed that dispersal limitation only caused moderate differentiation. The colonization of aphids is potentially a local event.

The populations of *A. gossypii* tended to be clustered into two groups that could be most easily understood in terms of genetic diversity. The high diversity group, represented by WXZ, had a higher percentage of polymorphic loci and a high Shannon index. The low diversity group, represented by LHC, had a lower percentage of polymorphic loci and a low Shannon index. The genetic diversity pattern resembled a patchwork with high and low diversity. The presence of population structure suggests that one or more evolutionary forces may be operating. These forces include limited migration, small local population sizes, restricted mating, and intrapopulation selection (Vaughn & Antolin, 1998). Dispersal affects the distribution of genetic diversity across space. Gene flow mediated by dispersal may drive or inhibit local adaptation, modulate the effect of genetic drift, and ultimately regulate population persistence (Mazzi & Dorn, 2012). Our results indicate that geographic distance is not a barrier to *A. gossypii* gene flow in this area.

### 4.2 Relatedness of genetic structure and landscape features

The GLM results demonstrate that genetic diversity is significantly associated with landscape features. Populations within landscapes containing large expanses of grassland tended to have higher genetic diversity than that of populations within areas with less grassland. However, orchard influenced the genetic diversity in a manner opposite to that of grassland. The greater the extent of the orchard land area was, the lower the genetic diversity of *A. gossypii* became. The orchard had a more relative importance than grassland to predict the genetic diversity.

Many landscape ecology studies suggest that special habitat or land cover affects insect colonization (Angelella et al., 2016; Dong, Ouyang, Lu, & Ge, 2015; Sivakoff, Rosenheim, Dutilleul, & Carrière, 2013). As landscape composition can affect population establishment and expansion, it could also affect the genetic structure. There are several possible explanations for the genetic diversity of *A. gossypii* populations being affected by land cover.

First, aphids can move from an unsuitable host to a more suitable habitat. The landscape composition, therefore, could influence aphid colonization. We did not measure the nearest distance of primary host plants from where aphids migrated. It has been reported that many plant species serve as primary hosts in North China, such as *Hibiscus syriacus* (hibiscus), *Punica granatum* (pomegranate), and *Zanthoxylum simulans* (Chinese prickly ash) (Wang et al., 2016). Moreover, these primary hosts are seldom planted in large areas. The widespread and differing species of primary hosts may influence the genetic structure of immigrant populations of *A. gossypii*. Some of the alate migrants may come from much farther places even representing anholocyclic populations that overwintered as adults. It is possible that these aphids are selecting particular habitat types based on visual cues (Favret & Voegtlin, 2001; Smith, 1969). Perhaps long‐distance migrant aphids make a habitat choice and then increase the genetic diversity by mixing with local aphids.

Second, the vegetation cover in the present study could also simply reflect the host plant distribution. Grassland had a positive effect on aphid genetic diversity possibly because grassland supplies abundant aphid resources. Many weeds in the Capparidaceae, Convolvulaceae, and Euphorbiaceae are hosts of *A. gossypii* (Brévault et al., 2008). The host distribution affects the *A. gossypii* genetics through selection and clonal reproduction. We cannot rule out the possibility that *A. gossypii* also overwinters on weeds. By the time of watermelon emergence, the aphids may disperse between watermelon and grassland. Grassland plants might also act as transitional hosts to bridge the time gap between spring watermelons and overwintering hosts. The orchard area, however, contains perennial, deciduous crops such as orchard tree species. Most of these habitats do not contain suitable host plants for *A. gossypii*. As the orchard area increases, the melon crop areas harboring *A. gossypii* decrease. The low genetic diversity of *A. gossypii* occurs when the landscape is dominated with orchard land.

At last, the landscape scales also had effects on the dispersal and colonization of *A. gossypii*. We examined the impact of landscape variables on genetic diversity at four spatial scales, with landscape radii varying from 0.5 to 3 km. Landscape features at a radius of 1 km surrounding focal watermelon fields explained the highest proportion of the variation in genetic diversity. Although we know relatively little about the movement of *A. gossypii*, it is possible that a landscape of this size encompasses their ecological neighborhood (Addicott et al., 1987), containing a diversity of habitats utilized by this species. Bahlai, Sikkema, Hallett, Newman, and Schaafsma (2010) used landscape parameters to identify fields at greatest risk of becoming colonized by soybean aphids (*Aphis glycines*). The present study about landscape genetics of aphids may help improve our understanding of colonization of *A. gossypii* and the corresponding spatial scale. However, the limited sample size and location may reduce the statistical power. Measuring the distribution of primary hosts could be a way to investigate aphid colonization. An alternative analysis strategy used in some landscape studies (e.g., Gardiner et al., 2009) which focuses on landscape diversity and combined explanatory variables could also be helpful. Future work should test the effect of year and in particular should examine host plant species, which could act as reservoirs.

## 5 CONCLUSIONS

Our study clearly supports the hypothesis that landscape features can affect the genetic variation of *A. gossypii*. The populations were structured not by geography but by their surrounding habitats. They were divided into two groups (high and low) based on genetic diversity. The results suggest that the colonization of watermelon by *A. gossypii* occurs locally and is affected by the surrounding habitats. More grassland in the landscape tends to favor the maintenance of genetic diversity. Orchard and their perennial plants reduced the genetic diversity of *A. gossypii*. The land cover, such as grassland and orchard, had a strongest effect at the spatial scale of 1 km. By identifying features of the landscape that surrounds fields and can affect these dynamics, growers can develop more efficient crop protection strategies relying on habitat manipulation.

## DATA ACCESSIBILITY

Raw 2b‐RAD sequence reads are available at NCBI PRJNA407691.

## CONFLICT OF INTEREST

The authors have declared that no competing interests exist.

## AUTHOR CONTRIBUTIONS

Z. Dong and Z. Zhang conceived and designed the study. Y. Li conducted sampling and isolated DNA. Z. Dong analyzed the data and prepared the manuscript.
